# Effect of muscone on anti-apoptotic ability of muskrat prostate primary cells

**DOI:** 10.1152/ajpregu.00068.2022

**Published:** 2022-08-15

**Authors:** Yu Zhang, Xuefei Zhao, Xin Man, Yue Ma, Wei Zhang, Yu Wang, Xing Lei, Suying Bai

**Affiliations:** ^1^College of Wildlife and Protected Area, Northeast Forestry University, Harbin, People’s Republic of China; ^2^National and Local Joint Engineering Laboratory for Freshwater Fish Breeding, Heilongjiang River Fisheries Research Institute, Chinese Academy of Fishery Sciences, Harbin, People’s Republic of China; ^3^Detecting Center of Wildlife, State Forestry and Grassland Administration, Harbin, People’s Republic of China

**Keywords:** apoptosis, muscone, muskrat (Ondatra zibethicus), primary culture, prostate

## Abstract

Muskrat is a small fur animal with a pair of scent glands that can secrete muskrat musk during breeding season. The consensus is muskrat musk functions as a pheromone, but we hypothesized it has a broader role. In previous research, we found the presence of muscone in muskrat musk. To study whether the muscone can affect the apoptosis of muskrat prostate, we carried out the following investigations. Primary muskrat prostate cells were cultured and treated with muscone. Then we drew cell proliferation curves by applying the CCK-8 and used TdT-mediated dUTP nick end labeling (TUNEL) to detect apoptosis. Levels of mRNA transcription and protein expression of *Bcl-2* as well as *Bax* were detected by qRT-PCR and the Western blot. Meanwhile, we collected tissue samples of muskrat prostates and froze sections to analyze the fluorescence signal intensity of BCL-2 and BAX via immunofluorescence. Under the treatment of 30 μmol/L muscone, the proliferation rate of the experimental group exceeded that of the control group, and the proportion of cells undergoing apoptosis was lower in the experimental group. The qRT-PCR and Western blot result showed that, in the experimental group, the ratio of *Bcl-2* to *Bax* mRNA transcription levels increased by 2.85 times and their corresponding protein expression ratio increased by 2.37 times (*P* < 0.05). Immunofluorescence results were consistent with the cell experiment’s results. The fluorescence signal intensity of BCL-2 was higher in the breeding season than nonbreeding season but vice versa for BAX. Based on these results, we speculate that the muscone could regulates prostate development by inhibiting apoptosis.

## INTRODUCTION

The muskrat (*Ondatra zibethicus*) belongs to the genus *Ondatra* (family Cricetidae). It is a small furry animal and widely distributed throughout the northern hemisphere. It is noted for its strong reproductive ability and rapid population growth and expansion, but because of habitat degradation (swamp ecosystem mainly) and other factors, muskrat populations are in decline (1–3).

The breeding season of muskrats in Northeast China beings in late March to early April, and the onset of their nonbreeding season is from late September to early October. Male muskrats have a pair of scent glands between their abdominal skin and muscles. During the breeding season, these glands can secrete viscous oily liquid with strong distinctive smell, namely, muskrat musk. The chemical composition of muskrat musk is very complex, including a variety of fatty acids and ketones (4). Previous studies have shown that muskrat musk has many physiological and pharmacological effects. For example, Chen et al. (5) found that an injection of muskrat musk can reduce the heart rate of anesthetized dogs and make mice more resistant to hypoxia. More recently, Lee et al. (6) found that muskrat musk confers a neuroprotective effect against transient focal cerebral ischemia via its oral administration to rats, perhaps achieved mechanistically by inhibiting the expression of *COX-2*. Work by Yuan et al. (7, 8) demonstrated that muskrat musk can inhibit cardiomyocyte apoptosis by regulating the protein expression of *Bcl-2* and *Bax*, and increasing the content of calcitonin gene-related peptide (CGRP) by reducing the release of plasma endothelin (ET), thereby improving myocardial ischemia and protecting the myocardium. Because of the pharmacological effects and potential economic value of muskrat musk, its rational utilization is of great significance in the field of medicine and ecological protection.

Muskrat’s scent glands are connected to the inner side of the external genital foreskin through a catheter. During the breeding season, male muskrats attract females by discharging muskrat musk to complete the mating process; that this muskrat musk functions as a pheromone is not in doubt and agreed upon. Yet, it remains unclear whether muskrat musk not only attracts females via an olfactory mechanism but also regulates the self-development of male muskrats. The development of muskrat’s scent glands is regulated by sex hormones and undergo predictable seasonal changes (9, 10). During the breeding season, the scent glands develop rapidly and become enlarged, and later on shrink rapidly to become smaller in the non-breeding season. Through early anatomical observations, we found that the development time of muskrat’s scent gland and testis coincided relatively well during the breeding season, which also supports the view that its scent gland development is regulated by sex hormones. The prostate volume of the male muskrat also changes periodically with the alternation of breeding and non-breeding seasons, but prostate development lags behind the scent gland and testis yet the prostate tissue is evidently highly developed during the reproductive period, which warrants attention. An early study showed that, after 13 days of muskrat-musk treatment, the seminal vesicle-prostate of juvenile mice was three times heavier than that of the control group, this proving that muskrat musk could significantly promote in mouse the development of its seminal vesicle-prostate (11). In previous research, we found the presence of muscone in muskrat musk (Supplemental Material; all Supplemental material is available at https://doi.org/10.5281/zenodo.6416470). Other research showed that muscone exists in the urine of male forest musk deer (*Moschus berezovskii*) obtained by bladder puncture, which suggests that even if the musk gland is an exocrine gland, muscone can still somehow enter this animal’s circulatory system (12). When compared with musk deer, the muscone in muskrat musk may still enter the internal environment of muskrats and conceivably also exert a physiological function. Whether the muscone in muskrat musk secreted by male muskrats during the breeding season affects their prostate development is worthy of in-depth study. We speculate that the muscone in muskrat musk will enter the internal body environment where it regulates the development of the male muskrat’s own prostates.

The B-cell lymphoma 2 (*Bcl-2*) gene family is among the most important in the study of apoptosis. Regulating mitochondrial membrane permeability, the endogenous pathway of apoptosis, is one of its prominent roles, and the ratio of *Bcl-2* and *Bax* gene expression is the key factor for determining whether cells will undergo apoptosis (13, 14). In this study, primary cultured muskrat prostate cells were used as the experimental model. Changes in cell proliferation and apoptosis were detected by CCK8 and TUNEL, expression of Bcl-2 and Bax genes’ mRNA and protein were detected by qRT-PCR and the Western blot, and the amount and localization of BCL-2 and BAX proteins in muskrat prostate tissues at different stages were detected by immunofluorescence, to investigate whether the muscone in muskrat musk could affect the prostate physiological development of male muskrat.

## MATERIALS AND METHODS

### Ethical Statement

All animals in this study were treated in accordance with China’s national legislative guidelines on animal welfare. The care and use of experimental animals was approved by the animal ethics committee of Northeast Forestry University (see Supplemental Material).

### Animals

The muskrats used in the research were purchased from the Wuchang muskrat farm, located in Heilongjiang Province, China (126.33°E, 44.04°N). The samples were collected in April (breeding season) and October (nonbreeding season). A total of six muskrats were used in this study, two of which were used for cell primary culture (April), and the rest were used for immunofluorescent detection (two in April and two in October). After the muskrats were taken to the laboratory, they were fed normally for 5 days to reduce their stress response. After the experimental samples were collected, the muskrats were euthanized by an air embolism.

### Primary Culture

Before the sampling procedure, muskrats were anesthetized with ether, and the sampling point on their body was disinfected with 75% ethanol. Under sterile conditions, the dorsal lobe of prostate was taken out and immersed in 75% ethanol for 30 s and then transferred to 1× PBS buffer (phosphate-buffered saline, Thermo Fisher, Waltham, MA). The external membrane was peeled off using tweezers and ophthalmic scissors under a stereomicroscope (Olympus, Tokyo, Japan), and the removed tissue was then divided into blocks, each ∼1 mm^3^ in size. These tissue blocks were washed five times with 1× PBS buffer, for 1 min each time, with any residual blood and external membrane removed. Next, each tissue block was put into a 15-mL sterile centrifuge tube, to which was added 5 mg/mL of type I collagenase (Sigma-Aldrich, St. Louis, MO) to immerse it; the tissue was digested for 1 h at a 37°C constant temperature on a shaking table (Olb-110x50, Tsinan, China) and then filtered through a 400-mesh sieve and the filtrate collected. After being centrifuged at 1,000 rpm for 5 min, the supernatant was removed, and the cells were resuspended and washed thrice with 1× PBS buffer. After the PBS buffer was removed, 5 mL of RPMI-1640 (Thermo Fisher, Waltham, MA) complete medium containing 10% fetal bovine serum (Thermo Fisher, Waltham, MA) was used to resuspend the cells. These were transferred to a T25 cell culture flask (Corning, New York) and placed in a CO_2_ incubator (Thermo Fisher, Waltham, MA) containing 5% CO_2_ and 95% air for constant-temperature culturing at 37°C, with the culture medium replaced every 48 h. When the cell confluence had reached more than 80%, subculturing was carried out. After the culture medium was discarded, 1× PBS buffer preheated to 37°C was added to wash the cells three times. Then, 2 mL of 0.25% trypsin (Thermo Fisher, Waltham, MA) preheated at 37°C was added, for a digestion lasting 3 min, during which time, the culture flask was shaken slowly to prompt the cells to fall off; the digestion was ended with serum, cell suspension moved into a centrifuge tube, and its supernatant removed after centrifugation at 1,000 rpm for 5 min. Finally, 1× PBS buffer was added to resuspend and clean the cells, followed by centrifuging again, repeated once, with the complete medium added to resuspend the cells, and subcultured according to 1:3. In addition to the experiment performed to detect the level of apoptosis by TdT-mediated dUTP nick end labeling (TUNEL), the cells used in this study came from 2 to 5 generations of muskrat prostate cells.

Muskrat prostate primary cells were treated with muscone (Vuco, Dalian, China) in DMSO. According to the preliminary experimental results, the cells of the experimental group (EG) were treated with 30 μmol/L muscone for 48 h; to the control group (CG) only, DMSO was added to render its final concentration equal to 0.1%. Six replicates were set for each EG group and CG group, of which three were used for real-time qPCR and three used for the Western blot.

### Cell Proliferation

The CCK-8 method (Cell Counting Kit-8, MCE) was used to detect the effect of muscone on the proliferation of muskrat prostate cells. All muskrat prostate cells used for proliferation detection were cultured for 10 days (i.e., 3rd generation). An EG group receiving 30 μmol/L of muscone and a CG group receiving 0.1% DMSO were set up. In addition, we set up two acellular blank groups, one containing 30 μmol/L muscone (labeled Ab) and the other containing 0.1% DMSO (labeled Ac), with three replicates for each. The 96-well plate was used for cell culturing, for which the cell density was adjusted to ca. 5,000 cells per well with the cell counting plate, and the total volume of each well was adjusted to 100 μL by adding the cell culture medium. A total of five time points were set, corresponding to 24, 48, 72, 96, and 120 h, each represented by three duplicated wells. The CCK-8 reagent was added (10 μL to each well) at 2 h before each time point. Next, the 96-well plate was put back into the incubator for 2 h. Finally, the absorbance of cells at a 450-nm wavelength was measured by an enzyme-labeling instrument (Thermo Fisher, Waltham, MA), and this data recorded to calculate the following:

$$\text{Cell survival rate }(\text{\%})=\frac{\text{EG group average OD value }-\text{ Ab group average OD value }}{\text{CG group average OD value }-\text{ Ac group average OD value }}\;\times \text{1}00$$

### TUNEL

The primary cells of muskrat prostate from 1 to 5 generations grew vigorously, exhibiting strong proliferation activity, with relatively few apoptotic cells present. Once subcultured to the fifth generation, the cell proliferation rate had declined significantly, whereas the proportion of apoptotic cells had increased significantly. Therefore, in detecting whether muscone could affect the apoptosis of muskrat prostate cells, we selected those muskrat prostate cells cultured for 35 days (i.e., 7th generation) to detect the apoptosis level by TUNEL. Muskrat prostate cells were inoculated into a six-well plate, corresponding to three replicates each in EG group and CG group. After 48 h, the cell culture medium was discarded, and the cells were washed three times with 2 mL of 1× PBS buffer. Next, the following steps were followed: add 200 μL of precooled 4% paraformaldehyde, fixation at 4°C for 2 min and apply 1× PBS buffer to wash the cells again; add 0.1% TritonX-100; and let it permeate for 5 min at 4°C. Then treat the positive control group with DNase I at 37°C for 30 min. After washing three times with 1× PBS buffer, add 3% H_2_O_2_ methanol and incubate cells in the dark for 5 min to remove the interference of endogenous peroxidase. After that, the cells were washed with 1× PBS buffer (three times, 5 min each time). A TUNEL apoptosis detection kit (Phygen, Fuzhou, China) was used to detect dead cells according to the manufacturer’s instructions. Hematoxylin was used to stain the nucleus. The number of positive cells in the EG and CG groups were counted (the total number of cells in each group was more than 2,000), and the apoptosis rate was calculated as follows:

$$\text{Celll apoptosis rate }(\text{\%})=\frac{\text{Numbers of apoptotic cells}}{\text{ Total numbers of cells }}\;\times \text{1}00$$

### RNA Isolation and cDNA First-Strand Synthesis

The cells were washed with 1× PBS buffer; 2 mL of 0.25% trypsin was added for a digestion lasting 3 min. The digestion was stopped with serum; after the cells fell off, they were transferred into the centrifuge tube. This was centrifuged at 1,000 rpm for 5 min and the ensuing supernatant was removed. The GeneJET RNA Purification Kit (Thermo Fisher, Waltham, MA) was used to extract total RNA by following the manufacturer’s instructions. To synthesize the first strand of cDNA, the PrimeScript RT reagent Kit gDNAEraser (Takara, Kyoto, Japan) was used according to instructions.

### Real-Time Fluorescence Quantitative PCR

The muskrat prostate cells *Bcl-2* and *Bax* genes transcription levels in EG group and CG group were detected by real-time qPCR. Their primers were designed used Primer Premier v6.0 software (Primer, ON, Canada). For the *Bcl-2* and *Bax* gene sequences, refer to the previous RNA-Seq results of our laboratory, for reference gene primers, see the sequences published by Lu et al. (9). The primer sequences are shown in Table 1. Each sample consisted of three duplicated wells and three groups of biological repeats. The instrument used was a CFX384 Touch Real-Time PCR Detection System (Bio-Rad). The 20-μL reaction system consisted of 10 μL of 2× SYBR Select Master Mix (Thermo Fisher, Waltham, MA), 0.5 μL of each forward and reverse primer (primer final concentration is 0.5 μmol/L), 2 μL of template cDNA, and 7 μL of ddH_2_O. The reaction program went as follows: 50°C for 2 min, 95°C for 2 min, denaturing at 95°C for 15 s, then annealed followed by an extension at 60°C for 1 min, after which fluorescence signals were collected. The denaturation and annealed extension were repeated in 40 cycles. After the reaction, whether nonspecific amplification occurred was judged by the melting curve. The reference gene *Actb* was used to normalize the target gene, and the relative difference in gene expression levels calculated by the 2^−△△CT^ method (15).

**Table 1.** Primer sequences used in the real-time qPCR

Primer	Primer Sequence (5′–3′)	Size of products (bp)
Bcl-2F	ACGGTGGTGGAGGAACTCTTCAG	166
Bcl-2R	TGTGCAGATGCCGGTTCAGGTA	
BaxF	GAACCATCATGGGCTGGACACTG	105
BaxR	TGGGCGTCCCGAAGTAGGAAAGG	
ActbF	TTGCTGATCCACATCTGCT	146
ActbR	GACAGGATGCAGAAGGAGAT	

### Protein Isolation and Western Blotting

The muskrat prostate cells were washed with 1× PBS buffer (3 times). These steps were then followed: add 2 mL 0.25% trypsin to digest until the cells fell off, terminate the digestion with serum, transfer the cells to a centrifuge tube, and then remove the supernatant after centrifugation at 1,000 rpm for 5 min; add 300 μL of RIPA lysis buffer (Solarbio, Beijing, China) containing 1 mmol/L of PMSF (phenylmethanesulfonyl fluoride), let it stand on ice for 10 min, and blow appropriately with a pipette to fully lyse the cells. Finally, the supernatant was collected and the total protein obtained by centrifugation at 12,000 rpm at 4°C for 5 min, using a cryocentrifuge (Thermo Fisher, Waltham, MA). To determine the concentration of protein in the samples, BCA protein quantitative Kit (Solarbio, Beijing, China) was used according to its instructions.

Next, the loading amount was adjusted to ∼30 μg according to the protein concentration. Each protein sample was fully mixed with 5 × SDS loading buffer (Solarbio, Beijing, China), and then all of them were incubated at 99°C for 5 min to fully denature the protein. These samples were processed by SDS-PAGE gel electrophoresis at a voltage of 150 V, for which the SDS-polyacrylamide gel (8%–20%) and predyed protein marker were purchased from Phygen (Fuzhou, China). The gel containing the target protein was cut off and the PVDF membrane in methanol, and then the protein was transferred onto a PVDF membrane under a constant current (200 mA) for 1 h. After the membrane was blocked with 5% BSA for 1 h, it was incubated respectively with primary antibodies of BCL-2, BAX, or β-ACTIN protein (1:3,000; respectively, Cat No. 66009-1-Ig, Cat No. 60178-1-Ig, and Cat No. 60267-1-Ig; Proteintech Group Chicago, IL) at 4°C overnight. The next day, the membrane was washed with 1× TBST buffer (tris buffered saline with Tween 20) three times (10 min each time), incubated for 1 h with an HRP-linked secondary antibody (Cat No. SA00001-1; Proteintech Group, Chicago, IL), and then washed again with 1× TBST buffer (three times, 10 min each time). An Ultra High Sensitivity ECL Kit (Enoch, Harbin, China) and Bio-Rad ChemiDoc XRS+ Gel Imaging System (Bio-Rad, CA) were used to detect the results. The optical density analysis was carried out in Image J software (https://mirror.imagej.net/); the BCL-2 and BAX proteins’ expression was normalized by the reference protein and their changed ratio calculated.

### Immunofluorescent Detection

The muskrat prostate tissue was fixed overnight in 4% paraformaldehyde phosphate buffer at 4°C. Before deriving their frozen sections, these steps were followed: wash with 1× PBS buffer three times (10 min each time), embed the tissue with Tissue-Tek OCT Compound (Tissue-Tek, Torrance, CA), and then use a Leica CM1900 cryostate (Leica, Wetzlar, Germany) to cut the respective tissue sections of the breeding and nonbreeding seasons; they had a thickness of 10 μm, were soaked in 1× PBS buffer (three times, 5 min each time), and blocked with 5% BSA for 1 h. The primary antibodies of the BCL-2 and BAX proteins (Cat No. SA00009-1 and Cat No. SA00003-1; Proteintech Group, Chicago, IL) were diluted with a blocking solution and incubated overnight at 4°C. The tissue sections were washed three times with 1× PBS buffer (10 min each time), after which they were incubated with a secondary antibody (Protentech group, Cat No. SA00009-1, Cat No. SA00003-1, Chicago, IL) for 1 h. After being incubated in DAPI (4′,6-diamidino-2-phenylindole) for 10 min, each tissue section was washed with 1× PBS buffer (three times, 10 min each time). The experimental results were observed under a fluorescence microscope (Nikon Eclipse Ti, Nikon Corporation, Japan), and the density and intensity of fluorescence signals between breeding and nonbreeding seasons were compared.

### Data Statistics and Analysis

SPSS 19.0 software (SPSS, Chicago, IL) was used for the statistical analysis. Significant differences in cell proliferation data were determined by a two-way ANOVA. Student’s *t* test was used to determine significant differences in the data of other response variables. Significance was set at *P* < 0.05. Data are presented here as the means ± standard error. Image J V1.8.0 software (Rawak Software Inc., Stuttgart, Germany) and GraphPad Prism 5 software (GraphPad Software, Inc., San Diego, CA) were used to draw the figures.

## RESULTS

### Primary Culture of Muskrat Prostate Cells

**Figure 1.**
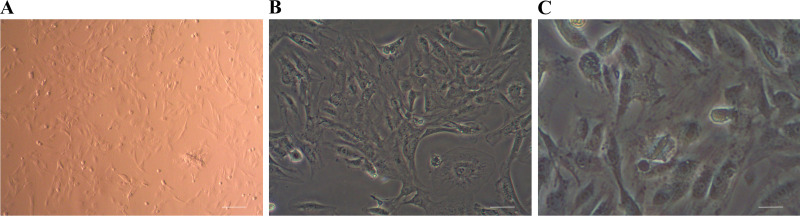
Primary cultured muskrat prostate cells. Scale bars of *A*–*C* are 50, 20, and 10 μm, respectively.

Muskrat prostate cells can be primarily cultured via conventional trypsin digestion. The newly digested and isolated cells are bright, spherical, and scattered individually, though occasionally cell clusters can be seen. Muskrat prostate cells adhered and extended relatively swiftly. When cultured for 120 min, the cells had basically adhered to the bottom of cell culture flask. After being cultured at 37°C for 24 h in 5% CO_2_, a variety of cells including epithelial cells and fibroblasts were seen under the microscope, arranged in a typical monolayer and paving stone-like mosaic. Evidently, stark differences characterize the morphology of different types of cells, in that epithelial cells are triangular or polygonal, having a large and round nucleus, and a rich cytoplasm. By contrast, fibroblasts are slender, spindle shaped, with a clear nucleus, being whirlpool like or vertically and horizontally arranged, as seen in Fig. 1, *A*–*C*.

Early on in the primary culture, the cells grew vigorously and proliferated rapidly. But with prolonged subculturing, after the fifth subculture, the cells displayed signs of senescence, by which time their proliferation rate had decreased, their cell volume was enlarged, and they eventually lost their original cell morphology. Under the microscope, the aged primary cells of muskrat prostate had vacuoles in the cytoplasm, the number of floating cells increased, and many cell fragments were apparent.

### Effect of Muscone on Cell Proliferation

**Figure 2.**
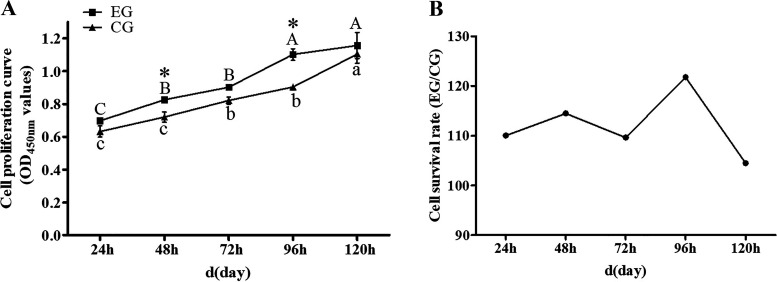
*A*: cell proliferation curve of the EG group and CG group. *B*: cell survival rate of the EG group vs. CG group. Different uppercase letters indicate significant differences within EG group, and different lowercase letters indicate significant differences within CG group. *Significant difference between EG group and CG group. CG, control group; EG, experimental group.

Cell proliferation was quantified using the CCK-8 method, and Fig. 2*A* shows the cell proliferation curves of EG group and CG group. At all five time points, the average absorbance (450 nm) of EG group surpassed that of the CG group (Table 2). Survival rates of cells treated with muscone for 1–5 days were respectively 110.07%, 114.53%, 109.63%, 121.76%, and 104.47%, which suggested that muscone can improve the proliferation rate of muskrat prostate cells (Fig. 2*B*).

**Table 2.** Cell survival rate in the EG group vs. CG group

	1 Day	2 Days	3 Days	4 Days	5 Days
OD450 mean value (EG-Ab)	0.6973	0.8260	0.9018	1.1014	1.1557
OD450 mean value (CG-Ac)	0.6335	0.7212	0.8226	0.9046	1.1062
Cell survival rate	110.07%	114.53%	109.63%	121.76%	104.47%

Ab, acellular blank group containing 30 μmol/L muscone; Ac, acellular blank group containing 0.1% DMSO; CG, control group; EG, experimental group.

### Effect of Muscone on Cell Apoptosis

**Figure 3.**
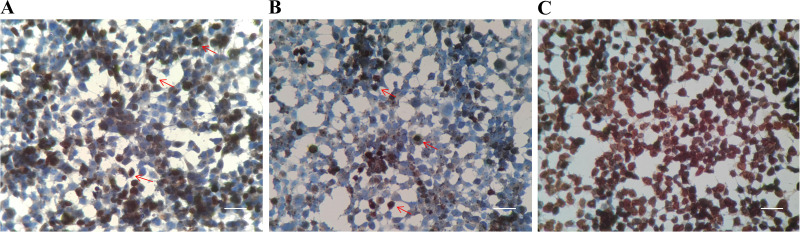
*A*: TdT-mediated dUTP nick end labeling (TUNEL) result for the CG group. *B*: TUNEL result for the EG group. *C*: positive control. Arrow pattern, positive cells in the state of apoptosis (scale bars, 40 μm). CG, control group; EG, experimental group.

The TUNEL results revealed that 48 h after muskrat prostate cells were treated with muscone, there were significantly fewer apoptotic cells in the EG group than the CG group (Fig. 3, *A*–*C*). The average proportion of apoptotic cells in the EG and CG groups was 24.25% and 41.10%, respectively (total number of cells > 2,000; Table 3). These results indicated that, at 48 h postmuscone treatment, nearly half of the CG group muskrat prostate cells were in a state of apoptosis, whereas the apoptotic proportion of muskrat prostate cells in the EG group was significantly lower (*P* < 0.05). This suggested muscone could significantly inhibit the apoptosis of senescence prostate cells in male muskrats.

**Table 3.** Proportion of apoptotic cells in the EG group vs. CG group

	EG-1	EG-2	EG-3	CG-1	CG-2	CG-3
Number of apoptotic cells	134	195	214	251	335	266
Total number of cells	675	783	764	688	731	649
Apoptosis proportion	19.85%	24.90%	28.01%	36.48%	45.83%	40.99%
Means ± standard deviation	24.25 ± 3.36%*			41.10 ± 3.82%		

*Significant difference between EG group and CG group. CG, control group; EG, experimental group.

### Effect of Muscone on Gene Transcription for *Bcl-2* and *Bax*

**Figure 4.**
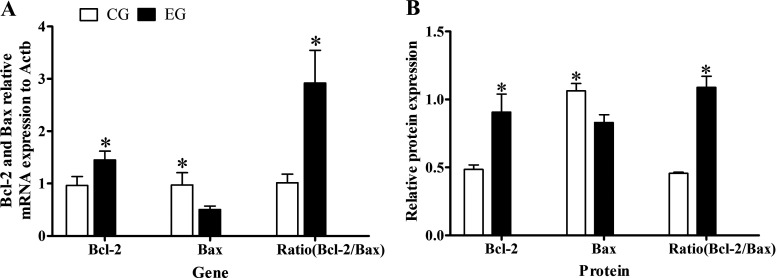
*A*: relative expression levels of *Bcl-2* and *Bax* mRNA in the EG group vs. CG group. *B*: relative expression of BCL*-*2 and BAX proteins in the EG group vs. CG group. *Significant difference between EG group and CG group. CG, control group; EG, experimental group.

The transcription level of genes encoding *Bcl-2* and *Bax* in EG and CG groups were detected by real-time qPCR. These results showed that the melting curves of *Bcl-2*, *Bax*, and reference gene for Actb were single peaks, implying no obvious nonspecific amplification; hence, the data were robust for use in the subsequent calculations and analyses. The relative expression of *Bcl-2* in the EG group was 1.51 times that in the CG group (*P* < 0.05), whereas that of *Bax* gene was about half that in the CG group (*P* < 0.05), such that the ratio of *Bcl-2* to *Bax* transcription levels was 2.85 times greater in the EG than CG group (*P* < 0.05; Fig. 4*A*). These real-time qPCR results demonstrated muscone was able to augment the transcription level of the *Bcl-2* gene in muskrat prostate cells while also reducing that of its *Bax* gene, thereby increasing the ratio between the two.

### Effect of Muscone on BCL-2 and BAX Proteins Expression

**Figure 5.**
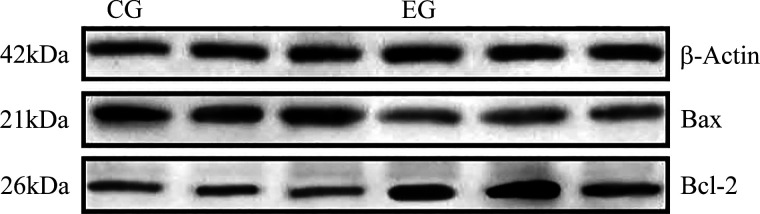
Western blotting results for BCL-2, BAX, and β-ACTIN proteins in the EG group and CG group. CG, control group; EG, experimental group.

The expression levels of BCL-2 and BAX proteins in the EG group and CG group were detected by the Western blot. These results appeared in Fig. 5 and showed that the expression of BCL-2 protein was 1.86 times higher in the EG than CG group (*P* < 0.05), whereas that of the BAX protein was 1.28 times higher in the CG than EG group (*P* < 0.05); accordingly, the ratio of BCL-2 to BAX in the EG group was 2.37 times that in the CG group (*P* < 0.05; Fig. 4*B*). These results showed that a muscone treatment of muskrat prostate cells for 48 h could significantly promote the expression of BCL-2 protein, inhibit the expression of BAX protein, and thus increase the ratio of their protein expression levels.

### Immunofluorescence Results of Muskrat Prostate Tissues

**Figure 6.**
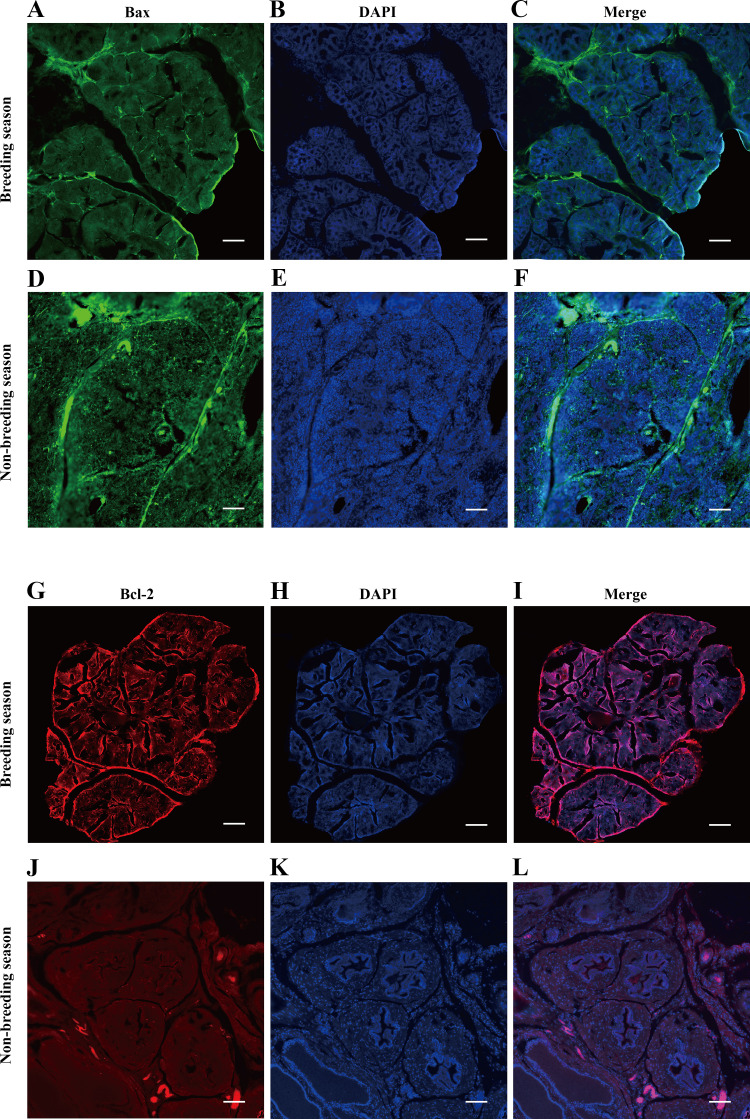
Immunofluorescence staining of BAX protein in the muskrat prostate of breeding season (*A*–*C*) and nonbreeding season (*D*–*F*). Immunofluorescence staining of BCL-2 protein in the muskrat prostate of breeding season (*G*–*I*) and nonbreeding season (*J*–*L*; scale bars, 100 μm).

Here, the density and intensity of the fluorescence signals of BCL-2 and BAX proteins in the muskrat’s prostate during its breeding season and nonbreeding season were analyzed by immunofluorescence techniques. These results uncovered BCL-2 and BAX proteins widely distributed in the prostate. However, the fluorescence signal intensity of the BCL-2 protein was significantly more pronounced in the breeding than nonbreeding season, but vice versa for the BAX protein (i.e., significantly lower in the breeding season than non-breeding season), as shown in Fig. 6.

## DISCUSSION

In this study, primary cells of muskrat prostate were used as an experimental model to study whether the muscone in muskrat musk can affect the self-development of male muskrats. The results for the primary culture of muskrat prostate cells showed that *1*) type I collagenase could effectively digest and disperse them, *2*) reasonable control of collagenase digestion time would not seriously damage them, and *3*) the cells could adhere and proliferate normally. This research showed that muskrat prostate cells can indeed be subcultured and cryopreserved according to conventional technical means. The cell model used in this research is a mixture of multiple cells, and we did not isolate and identify the cells. The reason being that our focus was on muscone’s overall effect on prostate cells, not on a single type of cell. Muskrat prostate cells are easy to obtain and culture and have the potential to become a cell model for muskrat physiological research. In future research, various cells of muskrat prostate tissue could be isolated and purified by flow cytometry, or other technical means, and their cell lines gradually established to lay a foundation for other muskrat studies.

There are many reports on the anti-apoptotic effect of muscone. Zhou et al. (16) found that muscone increased the expression level of *Bcl-2* and reduced that of the *Bax*, inhibited the apoptosis of flap tissue, and improved the survival rate of transplantation. Later, Dong et al. (17) found that muscone can inhibit the high glucose-induced apoptosis of rsc96 cells by regulating the Akt/mTOR signaling pathway. More recently, Wei et al. (18) showed that muscone could promote the activity of H9c2 cells, inhibit their apoptosis, and reduce injury to them caused by ischemia-reperfusion. However, whether the muscone in muskrat musk influences the apoptosis of muskrat prostate cells had not been studied until now. To do that, we treated muskrat prostate cells with muscone, detected their cell proliferation by CCK-8, and then detected their level of apoptosis by TUNEL. Our results showed that 1–5 days after treatment with muscone, the number of cells in EG group was greater than that in the CG group. The cell proliferation rate of the EG group tended to increase over the first 4 days, but it declined on the fifth day, perhaps due to cell quantity saturation having been reached. The TUNEL results showed that at 48 h since the muscone treatment, the average apoptosis rates of the EG and CG groups, respectively, were 26% and 43%, the proportion incurring apoptosis being significantly lower in the former than latter group, which proved that muscone could indeed inhibit the apoptosis of muskrat prostate cells. Furthermore, we speculate the enhanced cell proliferation rate of the EG group vis-à-vis the CG group is likely due less apoptotic cells. Through experimentation, we demonstrated that muscone can inhibit apoptosis, but whether muscone can directly promote the proliferation of muskrat prostate cells needs further research.

Apoptosis is an autonomous programmed cell death process that is paramount for maintaining the homeostasis of the environment in vivo (19–22). The *Bcl-2* gene family is the most extensively studied gene family in the mitochondrial apoptosis pathway, whose members represented by *Bcl-2* play a role in inhibiting apoptosis whereas another set of members represented by the *Bax* can promote apoptosis (23–29). The BCL-2 protein will polymerize with BAX protein to form a heterodimer that competitively inhibits the formation of a BAX homodimer, in this way preventing the destruction of mitochondrial membrane by BAX protein and the release of cytochrome C (30–33). In our study, we analyzed the effect of muscone on the expression of *Bcl-2* and *Bax* genes from two aspects, mRNA transcription (using real-time qPCR) and protein expression (using the Western blot). The results showed the altered pattern of mRNA transcription matching those of protein expression, which proved that muscone could increase *Bcl-2* gene expression and decrease *Bax* gene expression, thus increasing their ratio. That the detection results of *Bcl-2* and *Bax* genes’ expression corroborated the determined apoptosis rates at the molecular level provides compelling evidence that the anti-apoptotic effect of muscone on muskrat prostate cells may be achieved by increasing the expression ratio of *Bcl-2* to *Bax* gene. In our previous study, we found that muscone was able to promote the transcription level of *Bcl-2* gene, reduce the transcription level of *Bax* gene, and increase their ratio in HEK293T cells, findings consistent with the experimental results of the present study (33). Furthermore, in HEK293T cells, we found the *PI3K*, *NF-κB*, and *ERK* genes were differentially expressed, and because their signal transduction pathways are very important for the regulation of cell proliferation and apoptosis, it suggests a changed *Bcl-2* to *Bax* ratio under the muscone treatment may be related to these signal transduction pathways (34–36). Therefore, we speculate that the anti-apoptosis effect of muscone on muskrat prostate cells may arise via these signal transduction pathways.

Using the cell model for muskrat, we analyzed the anti-apoptotic effect of muscone on its prostate cells from multiple aspects, but attention should also be paid to whether the periodic changes of muskrat prostate development in its natural state are consistent with results based on the cell model. Hence, we detected the expression of BCL-2 and BAX proteins in the prostate tissue of muskrat during their breeding and nonbreeding seasons by immunofluorescence. Expression of the *Bcl-2* increased, whereas that of the *Bax* decreased in the prostate of muskrat in the breeding season compared with the nonbreeding season. These results for the natural prostate tissue of muskrat are consistent with the experimental results of the cell model, which suggests prostate development’s regulation may be similar to that of cell experiment; that is, the prostate tissue development of male muskrats may be affected by the muscone it secretes.

In another previous study, we sequenced the transcriptome of muskrat prostate tissue in the breeding season and nonbreeding season (37). Among the differentially expressed genes encountered, we screened a transcript named c75134_g2; after its sequence alignment, we found this transcript to be highly homologous to odor binding protein 2 (*OBP2*). A real-time qPCR analysis indicated its transcription level in the breeding season was more than 160 times that in nonbreeding season. According to the software-based prediction and analysis, the protein encoded by this transcript has a highly hydrophobic head and hydrophilic tail, whose middle part features an alternating hydrophilic and hydrophobic form, and a transmembrane structure. It likely functions as an odor molecule receptor.

**Figure 7.**
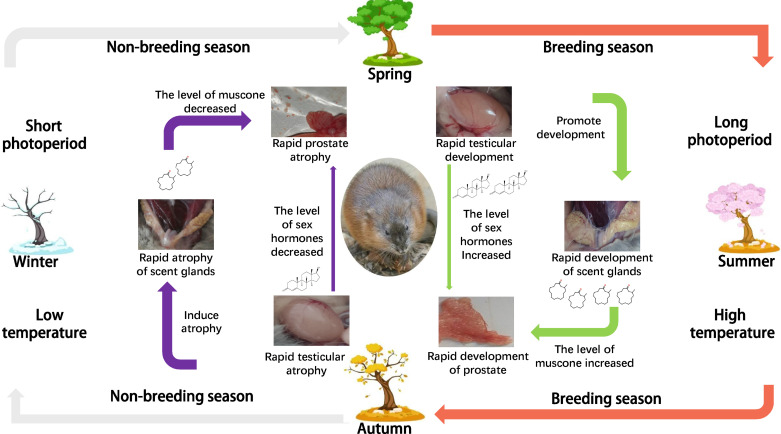
Sketch to show seasonal development of the prostate regulated by muscone.

Integrating those findings with the results reported here, we put forward a new view of how prostate development in muskrat is regulated. When the breeding season of muskrat is approaching, external stimuli such as temperature and light induce changes in a variety of biological signals. Among them, the rise of androgen level initiates the rapid development of seminal vesicles, prostate, scent glands, and other tissues and organs. The secretory epithelial cells of scent glands secrete muskrat musk, and some of the muscone in muskrat musk enters the internal circulation system. Prostate cells then synthesize a membrane protein (c75134_g2), whose function is to capture lipophilic odor molecules roaming freely in the circulatory system (i.e., muscone). Once the ligand and receptor are combined, a variety of downstream signal transduction pathways are activated, the expression levels of *Bcl-2* and *Bax* genes’ change, and the ratio of *Bcl-2* to *Bax* increases, consequently improving the anti-apoptotic ability of cells. Under the joint action of sex hormones and muscone, prostate tissue finally undergoes its rapid development. Before the nonbreeding season, the sex hormone level of muskrat is diminished; this renders its scent gland atrophied and the process of secreting muskrat musk declines to a halt. The reduction of sex hormone and muscone causes the prostate tissue of muskrat to shrink rapidly (Fig. 7).

### Perspectives and Significance

Muskrat musk is widely considered to play the role of a pheromone. Past research on muskrat musk mainly focused on its pharmacological effect and clinical application. This study instead focused on physiological effects of muskrat musk, by analyzing the effect of muscone on prostate development of male muskrats. We believe that the muscone in muskrat musk has a certain effect on the regulation of muskrat prostate development. Further related research into muskrat physiology is still in the nascent stage; however, because the detailed molecular mechanism responsible for the periodic development of muskrat’s prostate is not clear, pursuing fundamental in-depth studies is deemed worthwhile.

## DATA AVAILABILITY

The original contributions presented in the study are included in the article and Supplemental Material (https://doi.org/10.5281/zenodo.6978843). Further inquiries can be directed to the corresponding author.

## SUPPLEMENTAL DATA

10.5281/zenodo.6978843 Supplemental Material: https://doi.org/10.5281/zenodo.6978843.

## GRANTS

This study was funded by the wildlife protection and management project of the State Forestry and Grassland Administration (2019072, 2020070209).

## DISCLOSURES

No conflicts of interest, financial or otherwise, are declared by the authors.

## AUTHOR CONTRIBUTIONS

Y.Z., W.Z., and S.B. conceived and designed research; Y.Z., X.Z., X.M., Y.W., and X.L. performed experiments; Y.Z. and X.Z. analyzed data; Y.Z. interpreted results of experiments; Y.Z. prepared figures; Y.Z. drafted manuscript; Y.Z. and Y.M. edited and revised manuscript; S.B. approved final version of manuscript.
